# Nitrosyl- versus nitroxyl-cobalamin?

**DOI:** 10.1007/s00775-019-01658-1

**Published:** 2019-04-12

**Authors:** Justyna Polaczek, Łukasz Orzeł, Grażyna Stochel, Rudi van Eldik

**Affiliations:** 1 Faculty of Chemistry, Jagiellonian University, Gronostajowa 2, 30-387 Kraków, Poland; 2 Faculty of Chemistry, N. Copernicus University, Gagarina 7, 87-100 Toruń, Poland; 3 Department of Chemistry and Pharmacy, University of Erlangen-Nuremberg, Egerlandstrasse 1, 91058 Erlangen, Germany

**Keywords:** Nitric oxide, Nitrosyl, Nitroxyl, Cobalamin, Metal porphyrins, Non-heme complexes

## Abstract

The Commentary is in answer to the comment of a reader that objected against the use of the term ‘nitroxylcobalamin’ in two recent reports in JBC from our group. We use this opportunity to explain to the reader where this terminology originated from.

We received the following comment from a reader of JBIC concerning an apparent error made in two recent papers published by our group in JBIC [1, 2]:

‘The authors use the term nitroxyl to describe the coordination of NO to the cobalt of cobalamin. This term is not correct nomenclature. Nitroxyl or the IUPAC name azanone refers to the chemical compound HNO. The proper nomenclature of NO coordinated to cobalt is nitrosyl. JBIC should require its authors to use correct nomenclature when naming compounds. This needs to be addressed and corrected. This is a poor reflection on the journal as this should have been caught prior to publication.’

The Editor of JBIC consulted Nicolai Lehnert and George Richter-Addo about this statement, and here is what they said:

A criticism has been raised concerning the use of the term "nitroxyl" for the compound CblNO, which contains a coordinated NO to Cbl(II). While some authors have used the term "nitroxyl" for both HNO (IUPAC "azanone") and the NO^−^ anion, the CblNO compound is generally referred to in the literature as "nitrosyl-cobalamin". Consequently, for the CblNO compound, we strongly suggest to not refer to this compound as "nitroxyl-cobalamin", at least not without a proper explanation/justification. Otherwise, we feel that this causes unnecessary confusion for the reader. While an erratum is not explicitly required, we encourage you to provide one that uses the more appropriate term "nitrosyl", and this could be expanded to include the contribution of nitroxyl electronic character; e.g., "the nitrosyl CblNO which has substantial nitroxyl character”.

The Editor also consulted Peter C Ford after submission of our Commentary and here is what he said:

I have no objection to publication of the comments by van Eldik and colleagues. They make sense and explain their point of view, although I'm uncertain that they add more clarity to the controversy. The problem is that (as Enemark and Feltham recognized long ago) the bonding between NO and metal centers is highly delocalized, so that the assignment of formal oxidation states can be fraught with ambiguity. I personally agree with the view presented by Lehnert and Richter-Addo that metal–NO complexes can be given the generic term "metal nitrosyls" with no specific implication of oxidation states. For the specific case under discussion, the protonated form Cbl(HNO) would be the "nitroxyl" complex. I can appreciate the idea that reversible protonation of the CblCo^II^(NO) complex and the acute Co–N–O angle implies that the coordinated NO is reduced, and don't have a strong objection to the nomenclature used. However, the reversibility of the CblCo^II^ + NO reaction might be used to argue the other way. As chemists, we appreciate such ambiguity. I will offer another example that illustrates this issue of ambiguity. A few years ago, Elias Tfouni and I (and coworkers) [3] described the acid–base behaviors of the complexes Ru(salen)(NO)(OH_2_)^+^ (p*K*_a_ 4.5) and Ru(salen)(OH_2_)_2_^+^ (p*K*_a_ 5.9). As with other ruthenium nitrosyls, the former is typically referred to as NO^+^ coordinated to Ru(II), yet the H_2_O coordinated to that center is more acidic than in the latter case where the oxidation state is less equivocally Ru(III).

In answer to these comments, we would like to point out where our experimental evidence for nitroxyl-cobalamin originated from. In our first paper dealing with the reaction of aqua-cobalamin (CblOH_2_) with ^·^NO [4], we found that the reaction observed by other groups before was solely due to nitrite impurities in solution and no evidence for a reaction between CblOH_2_ and ^·^NO could be found. In a subsequent paper [5], we reported that reduced cobalamin, CblCo(II), rapidly and reversibly coordinated ^·^NO to form CblNO. We reported ^15^N-NMR evidence for the formation of a Co^III^–NO^−^ species, which was subsequently isolated [6, 7] and the crystal structure showed that NO^−^ is coordinated to Cbl(III) with a bond angle of 117°–121°, i.e. iso-electronic with dioxygen. At this point we referred to the CblNO complex as nitroxyl-cobalamin in order to distinguish between Co^II^–^·^NO (nitrosyl complex) and Co^III^–NO^−^ (nitroxyl complex). Ever since, we always referred to the CblNO complex as the nitroxyl complex. Recently, the p*K*_a_ of Cbl(HNO) was found to be 7.75 ± 0.03 [8], which is close to the value of 7.7 reported for [Fe^II^(CN)_5_HNO]^3−^ [9] and a reasonable ca. 4 units lower than the p*K*_a_(HNO) of 11.4 [10]. Thus, Cbl(HNO) can deprotonate under biological pH conditions to form Cbl(NO^−^), such that both complexes can be described as nitrosyl-cobalamin that has substantial nitroxyl character.

The confusion with reactions between metal ions/complexes and nitric oxide (^·^NO) originates from the fact that ^·^NO is a non-innocent, redox-active ligand and can formally coordinate as ^·^NO (nitric oxide), NO^−^(nitroxyl anion) or NO^+^ (nitrosonium cation) via redox reactions with the metal center. For a detailed treatment of NO^x^ chemistry, see references [11–16]. In this respect, we studied many Fe(III) porphyrin systems in which NO coordinates as Fe^II^–NO^+^, i.e. iso-electronic with CO and CN^−^, and binds linearly to the metal center [17–19]. The same was reported for the coordination of ^·^NO to cytochrome P450_cam_ in the absence (resting state) and presence of camphor as substrate, during which Fe^II^–NO^+^ is formed in both cases [20, 21]. These complexes were referred to as nitrosyl complexes and not as nitrosonium complexes. Also in the case of non-heme systems with Fe^III^–NO^−^ character [22, 23], we referred to them as nitrosyl complexes due to our uncertainty in terms of the nitroxyl character in the absence of the structural data for the nitroxyl-cobalamin complex published a few years later [6, 7].

Personally, as experimental coordination chemists, our goal was always to understand the coordination chemistry in terms of the formal oxidation state of the metal center. A part of the problem may further come from the use of the Enemark and Feltham notation {M–NO}^*n*^, where *n* equals the number of d electrons on the metal center plus one for the unpaired electron on ^·^NO [24]. In this notation no differentiation is made in terms of the oxidation state of the metal center and the electronic nature of coordinated ^·^NO.

More recently, the application of DFT and other computational techniques have been used to study the electronic character of metal–NO bonds in more detail [25–29]. From such studies partial or full charge transfer between the metal center and the ^·^NO ligand can occur in both directions to introduce partial NO^+^ or NO^−^ character depending on the electronic charge distribution in the metal–NO bond [20, 21]. Therefore, many M–NO bonds are presently described in terms of resonance structures such as Fe^III^–^3^NO^−^ ↔ Fe^II^–^·^NO, etc.

Finally, we understand the criticism of the reader and trust that the reader will now have a better understanding for our preference to refer to coordinated HNO and NO^−^ in the case of cobalamin as nitroxyl based on work done almost 20 years ago before computational studies were developed as far as they are today.
